# Associations of fish and meat intake with iron and anaemia in Malawian children

**DOI:** 10.1111/mcn.13622

**Published:** 2024-01-12

**Authors:** E. Rochelle Werner, Charles D. Arnold, Bess L. Caswell, Lora L. Iannotti, Kenneth M. Maleta, Christine P. Stewart

**Affiliations:** ^1^ Institute for Global Nutrition University of California, Davis Davis California USA; ^2^ Department of Global Health, Rollins School of Public Health Emory University Atlanta Georgia USA; ^3^ U.S. Department of Agriculture Western Human Nutrition Research Center Davis California USA; ^4^ E3 Nutrition Lab Washington University in St. Louis St. Louis Missouri USA; ^5^ School of Global and Public Health Kamuzu University of Health Sciences Blantyre Malawi

**Keywords:** anaemia, ferritin, infant and young child feeding, iron deficiency, small indigenous fish

## Abstract

Animal flesh foods are rich in bioavailable iron but infrequently consumed by young children. We aimed to determine whether flesh food intake was associated with iron and anaemia status among 585 Malawian infants enroled in a 6‐month egg‐feeding trial. The percentage of days of small fish, large fish and meat consumption were assessed through weekly 7‐day animal‐source food screeners. Grams of intake were assessed through 24‐h recalls conducted at 6–9, 9–12 and 12–15 months of age. Plasma ferritin, soluble transferrin receptor (sTfR) and haemoglobin concentrations were measured at 6–9 and 12–15 months of age. Iron biomarkers were adjusted for inflammation during analysis. At enrolment, each flesh food category was consumed by <5% of children in the past 24 h. Over the next 6 months, small fish, large fish and meat were consumed on 25%, 8% and 6% of days, respectively, with mean usual intakes of <5 g/day. More frequent small fish consumption was associated with lower sTfR (geometric mean ratio [95% CI]: 0.98 mg/L [0.96, 1.00] per 10 percentage point difference) but not ferritin (1.03 µg/L [0.98, 1.07]) or haemoglobin (1.01 g/dL [1.00, 1.01]). Large fish consumption was associated with higher anaemia (prevalence ratio [95% CI]: 1.09 [1.01, 1.19]) and lower iron deficiency (0.96 [0.93, 1.00]) prevalence. Gram intakes of flesh food categories were not associated with any iron or anaemia indicators. Small fish were a primary contributor to flesh food intake in this cohort of Malawian children, although usual portions were small. Fish was associated with modest improvements to iron status, but meat was too infrequent to be associated with anaemia and iron deficiency.

## Key messages

- Small fish was the most frequently consumed flesh food among young children: 4% at enrolment and 40% at the 6‐month follow‐up. On average, children consumed small fish once every 4 days.
- Despite small usual portions of about 5 g/day, frequent consumption of small fish was associated with moderate improvements in iron status.
- Large fish consumption was associated with lower iron deficiency but not with mean plasma ferritin or sTfR. Meat was infrequently consumed and not associated with any iron or anaemia measures.
- Reducing the burden of iron deficiency among young Malawian children may require provision of supplemental dietary iron, improved complementary feeding practices, addressing underlying barriers to flesh food consumption and controlling inflammation and infectious diseases.

## Graphical abstract

Small fish were the most frequently consumed flesh food among Malawian children aged 6–15 months old. Despite small usual intakes of about 5 g/day, frequency of small fish consumption was associated with moderate improvements to iron status. Large fish and meat were infrequently consumed and had inconsistent or no associations, respectively, with iron or anaemia.

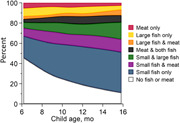

## 1 INTRODUCTION

Infants and young children are at high risk of iron deficiency due to low iron stores from birth (PAHO/WHO, 2003), infrequent intake of iron‐rich foods (White et al., 2017) and low bioavailability of iron in plant‐based diets (Zimmermann & Hurrell, 2007). In Malawi, over 40% of children at 6–23 months of age suffer from iron deficiency (National Statistical Office (NSO) et al., 2017). Diets typically consist of a maize‐based porridge along with some legumes and dark leafy greens, which contain compounds like phytates, polyphenols, oxalates and tannins that impede iron absorption (Lynch et al., 2018; The Institute of Medicine, 2001; Zimmermann et al., 2005). Flesh foods, including meat, organ meat or fish, have highly bioavailable haem iron and can also improve absorption of nonheme iron from other foods consumed within the same meal (Lynch et al., 2018; Michaelsen et al., 2009; The Institute of Medicine, 2001; Zimmermann & Hurrell, 2007). However, flesh foods are infrequently consumed and were reported for only 32% of children aged 6–23 months old through 24‐h dietary recalls in the 2015–2016 Malawi Demographic and Health Survey (National Statistical Office NSO Malawi, & ICF, 2017).

In 2018–2019, we conducted a randomised controlled trial: providing one egg per day to infants aged 6–9 months at enrolment in the Mangochi District of Malawi (Stewart et al., 2019). At the 6‐month follow‐up visit, mineral deficiencies and inadequate intakes were highly prevalent among children 12–15 months old: 61% were zinc deficient (Perez‐Plazola et al., 2023), 89% were iron deficient (inflammation‐adjusted ferritin <12 µg/L or soluble transferrin receptor [sTfR] >8.3 mg/L) (Werner et al., 2022) and ≥98% of children had inadequate intake of dietary iron (Caswell et al., 2021). Overall, the mean total dietary intake of iron from breast milk and complementary foods at enrolment was 1.9 mg/day (17% recommended dietary allowance) for infants 6–12 months [The Institute of Medicine, 2001]) and remained low at 3‐month (control: 2.5 mg/day; egg: 2.6 mg/day) and 6‐month follow‐ups (control: 2.8 mg/day; egg: 3.0 mg/day) (Caswell et al., 2021). However, the percentage of dietary recalls reporting consumption of iron‐rich flesh foods increased over the study period (enrolment [6–9 months]: 23% in the control group, 34% in the egg group; 3‐month follow‐up [9–12 months]: 68% in control, 65% in egg; and 6‐month follow‐up [12–15 months]: 67% in control, 72% in egg) (Lutter et al., 2021). Thus, this cohort consumed flesh foods at a higher frequency than the national average, yet still had a very high prevalence of iron deficiency.

The objectives of this analysis were twofold. First, we aimed to quantify total versus bioavailable dietary iron intake by food source among complementary foods reported in 24‐h dietary recalls of Malawian children enroled in the Mazira Project. Second, we aimed to determine whether the frequency and portions of small fish, large fish and meat reported through weekly animal‐source food screeners and quarterly 24‐h dietary recalls, respectively, were associated with higher ferritin, lower sTfR, higher haemoglobin and lower prevalence of anaemia, iron deficiency and iron deficiency anaemia.

## 2 METHODS

### 2.1 Study design and participants

The Mazira trial (clinical trials registry, NCT03385252) was conducted in the Mangochi District of Malawi between February 2018 and January 2019. The study area was located along the southeastern shore of Lake Malawi, where fishing and farming are the predominant occupations and most children are breastfed through 23 months (National Statistical Office NSO Malawi, & ICF, 2017). This region is also known to have higher burdens of anaemia among pregnant women (43%–45%; Jorgensen et al., 2018), child stunting (33%–38% of 18‐month‐old children; Ashorn et al., 2015) and micronutrient deficiencies (11%–15% vitamin A deficiency in 6‐month‐old infants; Haskell et al., 2021) than the national average (National Statistical Office NSO Malawi, & ICF, 2017). From previous research in these communities, children aged 6–18 months had morbidity symptoms on 22%–24% of days per caregiver report and five nonscheduled visits to any health facility per child per year of follow‐up, predominantly with diagnoses of malaria and acute respiratory infections (Bendabenda et al., 2016).

Children were individually randomised to receive either one egg per day or continue their usual diet for 6 months. A total of 660 children aged 6–9 months were enroled among age‐eligible children identified through household listings in the health service catchment areas of the Lungwena Health Centre and St. Martins' Hospital, Malindi (Stewart et al., 2019). Children were screened for the following eligibility criteria: singleton birth, intent to reside in the catchment area for the duration of the study, mid‐upper arm circumference ≥12.5 cm, haemoglobin >5 g/dL and the absence of bipedal oedema, egg allergy, recent hospitalisation or other morbidities that may affect growth or development. Children who screened positive for malaria, wasting, severe anaemia, bipedal oedema or other symptoms indicative of need for urgent care were referred to local health facilities.

Interviewers administered demographic and socioeconomic surveys including household assets and food insecurity (Coates et al., 2007) at enrolment, and they conducted anthropometric and developmental assessments, blood draws and 24‐h dietary recalls at the clinic during enrolment, when children were 6–9 months old, and endline visits, when children were 12–15 months old. At midline visits (9–12 months of age), anthropometric assessments and 24‐h dietary recalls were completed through home visits. All households were visited twice weekly from the third week after enrolment through the end of the 6‐month study period. During the second visit each week, caregivers responded to a screener regarding their child's intake of animal‐source foods over the past 7 days.

For description of dietary iron intake in the study setting, iron from complementary foods was totalled by food group categories for only the children in the control group (*N* = 329 at enrolment; 304 at midline; 301 at endline). However, for analytical models, this analysis included children from both group assignments with haemoglobin at endline. Children from both study arms were combined because flesh food consumption, iron intake and iron and anaemia biomarkers did not differ between the egg intervention and control arms (Caswell et al., 2021; Lutter et al., 2021; Werner et al., 2022). Study arms did not differ in missingness, and comparisons of children with complete versus missing data have been published previously (Werner et al., 2022). Compared to children missing haemoglobin at endline, children included in this analytical sample had mothers with higher education, literacy and employment in the service industry; lived in homes constructed with higher quality materials and closer to the Malindi Health Centre and had less household food insecurity.

### 2.2 Blood collection and laboratory analysis

Nurses collected venous blood from children at enrolment and endline into 5 mL lithium heparin collection tubes. Haemoglobin in whole blood was measured using Hemocue Hb 201 devices (HemoCue Inc.), and the presence of malarial antigens was measured using a rapid diagnostic test kit (SD Bioline Malaria Ag P.f/Pan; Abbott Diagnostics). Blood collection tubes were wrapped in foil, immediately placed on ice and centrifuged at 1040*g* for 15 min at room temperature. Laboratory technicians placed plasma into aliquots and temporarily stored them in a −20°C freezer on‐site. At the end of each day, samples were transported on ice to a −80°C freezer for storage and later shipped on dry ice by international courier to UC Davis. For each child who provided a minimum plasma volume of 450 µL, an aliquot of 50–75 µL plasma was shipped on dry ice to the VitMin Laboratory in Germany for analysis. Ferritin, sTfR, c‐reactive protein (CRP) and α−1‐glyoprotein (AGP) were analysed by combined sandwich ELISA techniques for each sample in a single well (Erhardt et al., 2004). For quality assurance, a subset of 10% of samples were rerun, and each tray was run with replicate controls of pooled plasma. The coefficient of variation of the controls for each index is as follows: ferritin (2.3%), sTfR (3.6%), CRP (5.8%) and AGP (8.1%).

### 2.3 Dietary assessments

Multiple‐pass 24‐h dietary recalls were adapted for tablet‐based administration with food lists specific for Malawi and administered at enrolment, midline and endline visits. Additionally, study staff conducted one to two repeat dietary recalls per time point through home visits among a subset of 200 participants. Ingredient‐level food and nutrient intakes were estimated from foods and drinks reported in the 24‐h dietary recalls using study‐specific recipes, portion conversions and food composition tables. Details of the Mazira Project 24‐h dietary recall data collection and processing have been reported previously (Caswell et al., 2021).

For each 24‐h dietary recall, observed mean iron intakes from complementary foods were summed by food group categories (World Health Organization & United Nations Children's Fund (UNICEF), 2021), with the addition of snack foods (Lutter et al., 2021) and subcategories for flesh foods (small fish, large fish, meat and organ meat). Small fish were differentiated from large fish by likelihood to consume the small fish whole (e.g., *bonya* [juvenile *usipa*], *usipa* [*Engraulicypris sardella*; freshwater sardine] and *kambuzi* fishes [assorted species of small fish, potentially including juvenile large fish]) or as a fillet from larger fish species (e.g., tilapia, catfish). Meat consisted of chicken, duck and goat meat. For each time point, observed mean iron intakes were compared to the estimated average requirement (EAR), a measure of dietary intake that is adequate for 50% of the population. Bioavailable iron was estimated by applying correction factors listed in Supporting Information: Table 1. Mean bioavailability‐adjusted intakes were compared to estimated iron needs (The Institute of Medicine, 2001), which reflect physiological need. Subsequently, the food groups that primarily contributed to estimated iron intakes were compared between both assessment methods to illustrate the relative contributions of flesh foods towards meeting iron needs. For analytical models, dietary intakes (g) were summed by flesh food category for each recall. Organ meats were rarely reported and were thus categorised with meat for analytical models. Large and small fish were analysed separately because iron content may be higher in small fish from consumption of internal organs (Thilsted et al., 2016) and enough children reported large fish to create a distinct category.

The weekly animal‐source food (ASF) screener asked caregivers to recall the number of days children consumed small fish, large fish, meat, eggs or milk over the past 7 days. Data from ASF screeners were cleaned to eliminate duplicate entries and select recalls administered on days closest to their scheduled weekly visit that did not overlap with the preceding 7‐day recall period by more than 1 day. Frequency of egg and milk consumption was not assessed due to their lower iron content, infrequent milk consumption (Lutter et al., 2021) and previously reported analyses of the egg intervention (Stewart et al., 2019; Werner et al., 2022). The percent of days small fish, large fish or meat were consumed was calculated for each child using screeners available over the full 6‐month study period as well as the last 3 months of the study period to reflect recent intake and lifespan of red blood cells. The frequency of any small fish, large fish and meat consumption over the same time periods was summed by the percent of weeks since ASF screeners did not specify the days of the week each flesh food was consumed.

### 2.4 Statistical analysis

A statistical analysis plan was prespecified and posted online (https://osf.io/vfrg7). All data cleaning, variable creation and analysis for frequency of ASF consumption from the weekly screeners were performed using deidentified data in Stata, version 15 (StataCorp, 2017). Analysis of usual intakes from 24‐h dietary recalls was conducted in SAS version 9.4 (SAS Institute Inc, 2013).

The outcomes of this study were mean concentrations of plasma ferritin, plasma sTfR and haemoglobin; anaemia (haemoglobin <11 g/dL); iron deficiency (ferritin <12 µg/L or sTfR >8.3 mg/L) and iron deficiency anaemia at endline. Ferritin, sTfR and iron deficiency were adjusted for inflammation using the regression approach recommended by the Biomarkers Reflecting Inflammation and Nutritional Determinants of Anaemia (BRINDA) working group (Namaste et al., 2017; Rohner et al., 2017). β‐coefficients for regression models were estimated separately for the enrolment and endline iron biomarkers and applied to any observations for which AGP > 0.59 g/L and/or CRP > 0.10 mg/L (Namaste et al., 2017). For inflammation adjustment and analytical models performed on the log scale, biomarker values below the lower limit of detection were replaced with half of the limit of detection, and values above the upper limit of detection were replaced with the maximum observed values.

Using the 24‐h dietary recall data, we estimated the usual grams of small fish, large fish and meat consumed at the midline and endline visits using the national cancer institute (NCI) method to account for within‐person variation in reported intake (National Cancer Institute, 2022). Briefly, we used the SAS macro, *MIXTRAN* (v.2.21), to estimate population‐level parameters for the amount and probability of consumption that were required to run the *DISTRIB* (v.2.2) macro, which estimated the median and percentiles of usual intake for small fish, large fish and meat for each time point (Luo et al., 2022). Afterwards, we replaced values for 0 g intake of flesh foods with one‐quarter of the minimum observed value for this analysis since predicted usual intake values were analysed on a Box‐Cox transformed scale. Subsequently, we used the *INDIVINT* macro (v.2.3) to examine the association between predicted usual intakes of small fish, large fish and meat at midline and endline with iron and anaemia at endline through regression models (Kipnis et al., 2009). This procedure was repeated for at least 200 (and up to 300) successfully converged bootstrapped iterations of each regression model to obtain standard errors. These models assessed associations of usual intakes of flesh foods at times corresponding to the beginning and end of production of red blood cells that may be in circulation at endline.

We used linear regression models to examine the difference in means of log‐transformed ferritin, sTfR and haemoglobin at endline for each 10% higher frequency of flesh food intakes from ASF screeners and 1 g higher usual intakes of small fish, large fish and meat from 24‐h dietary recalls. We used modified Poisson regression models with a log link to estimate prevalence ratios of anaemia, iron deficiency and iron deficiency anaemia for each 10% higher frequency and 1 g higher usual intake of flesh foods. All analyses controlled for baseline measures of the outcome. Fully adjusted models controlled for child sex, age, malaria, caregiver report of illness on the day of the dietary recall and month of assessment. Additionally, we examined outcomes for bivariate relationships with the following variables: health centre, maternal education, household assets, number of children under 5 years and minutes between blood draw and aliquot processing. Variables demonstrating a bivariate relationship with the outcomes (*p* < 0.1) were included as additional covariates in analytical models.

For sensitivity analyses, we also examined results by carrying forward the last observation to impute missing ASF screener data as well as dropping participants who completed less than 50% of the weekly screeners. To better understand the relationship of large fish consumption over 6 months with anaemia and iron deficiency, we conducted exploratory post hoc analyses adjusting for differences in demographic characteristics between participants in the upper and lower quartile of large fish consumption over 6 months, as well as variables associated with nonnutritional anaemia.

### 2.5 Ethical statement

Study staff informed caregivers of the research purpose, activities and their rights. Caregivers had opportunity to ask questions in a group setting as well as privately. Caregivers provided written, informed consent by signature or thumb print to enrol their child in the study and allow collected data and samples to be used for future research. All procedures were reviewed and approved by the Institutional Review Board at the University of California, Davis (UC Davis), and the Research Ethics Committee at the University of Malawi College of Medicine.

## 3 RESULTS

### 3.1 Description of children

Of the 660 enroled children, this analysis included 585 children with haemoglobin at endline (Supporting Information: Figure 1). At enrolment, small fish, large fish, meat, milk, organs and eggs were each consumed by less than 5% of children on the day preceding the recall (Supporting Information: Table 2). Approximately, one‐fourth of infants consumed vitamin‐A‐rich fruits and vegetables; one‐third consumed legumes, nuts or seeds and one‐half consumed other fruits and vegetables, such as tomatoes and onions. At enrolment, 4% of infants' caregivers refused consent for the blood draw, 8% of infants were unable to complete the blood draw and 9% of infants did not provide sufficient volume for analysis of iron. This sample of children had a high burden of inflammation (62% with CRP > 5 mg/L or AGP > 1 g/L), malaria (13%), anaemia (61%) and inflammation‐adjusted iron deficiency (77%) at enrolment (Supporting Information: Table 2). The prevalence of these conditions remained high at the 6‐month endline assessment (51% inflammation, 6% malaria, 43% anaemia and 89% iron deficiency; Supporting Information: Table 3). The average number of weekly ASF screeners completed per child included in the analysis was 21 ± 4.

### 3.2 Intake of iron and flesh foods

**Figure 1:**
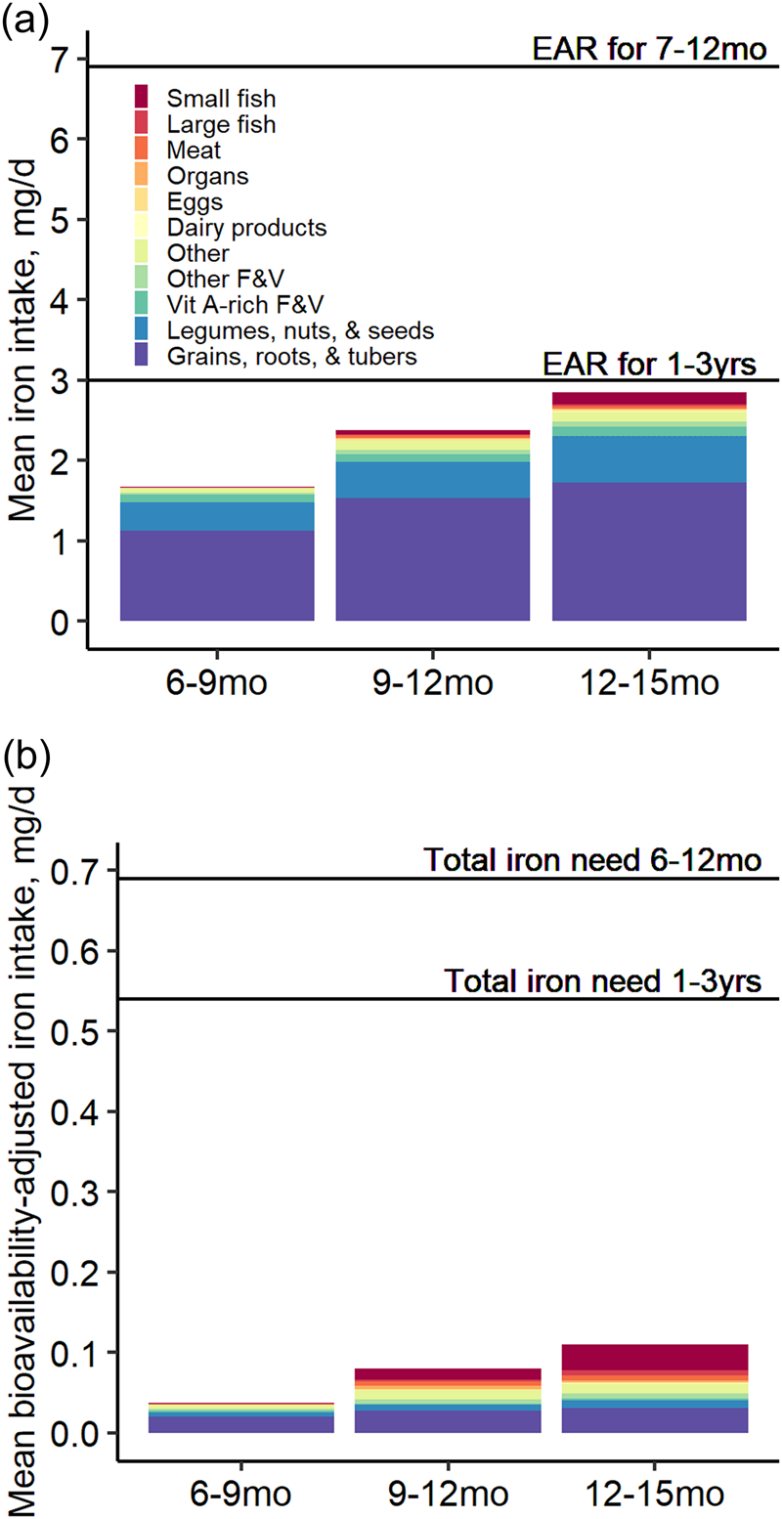
Mean (a) observed and (b) bioavailability‐adjusted iron intake by dietary source among children 6–15 months old in Mangochi, Malawi, 2018–2019. Children assigned to the control group provided 24‐h dietary recalls at 6–9 months (*n* = 329), 9–12 months (*n* = 304) and 12–15 months (*n* = 301). Correction factors listed in Supporting Information: Table 1 were applied to adjust observed dietary iron for absorption. Median total iron need for infants aged 6–12 months and 1–3‐year‐old children is 0.69 and 0.54 mg/day (The Institute of Medicine, 2001), respectively. EAR, estimated average requirement; F&V, fruits and vegetables.

Compared to the EAR of 6.9 mg/d for 7–12 months and 3.0 mg/d for 1–3 years, the observed mean dietary intake of iron from complementary foods was low: 1.7 mg at enrolment, 2.4 mg at midline and 2.9 mg at endline (Figure 1a). Grains, roots and tubers were the primary source of total dietary iron from complementary foods (Figure 1a). Few children consumed iron‐fortified infant porridge (4.0% at enrolment, 2.5% at midline and 2.2% at endline). Among these consumers, fortified infant porridge provided a median of about 0.40 mg iron at both enrolment and midline and 0.46 mg iron at endline or 11%–18% of children's total dietary iron intakes by time point. The estimated bioavailable iron was low at each time point (Figure 1b). However, at endline, flesh foods provided 43% of estimated bioavailable iron, and the average portion of small fish provided bioavailable iron similar to that of the food group of grains, roots and tubers (Figure 1b).

**Figure 2:**
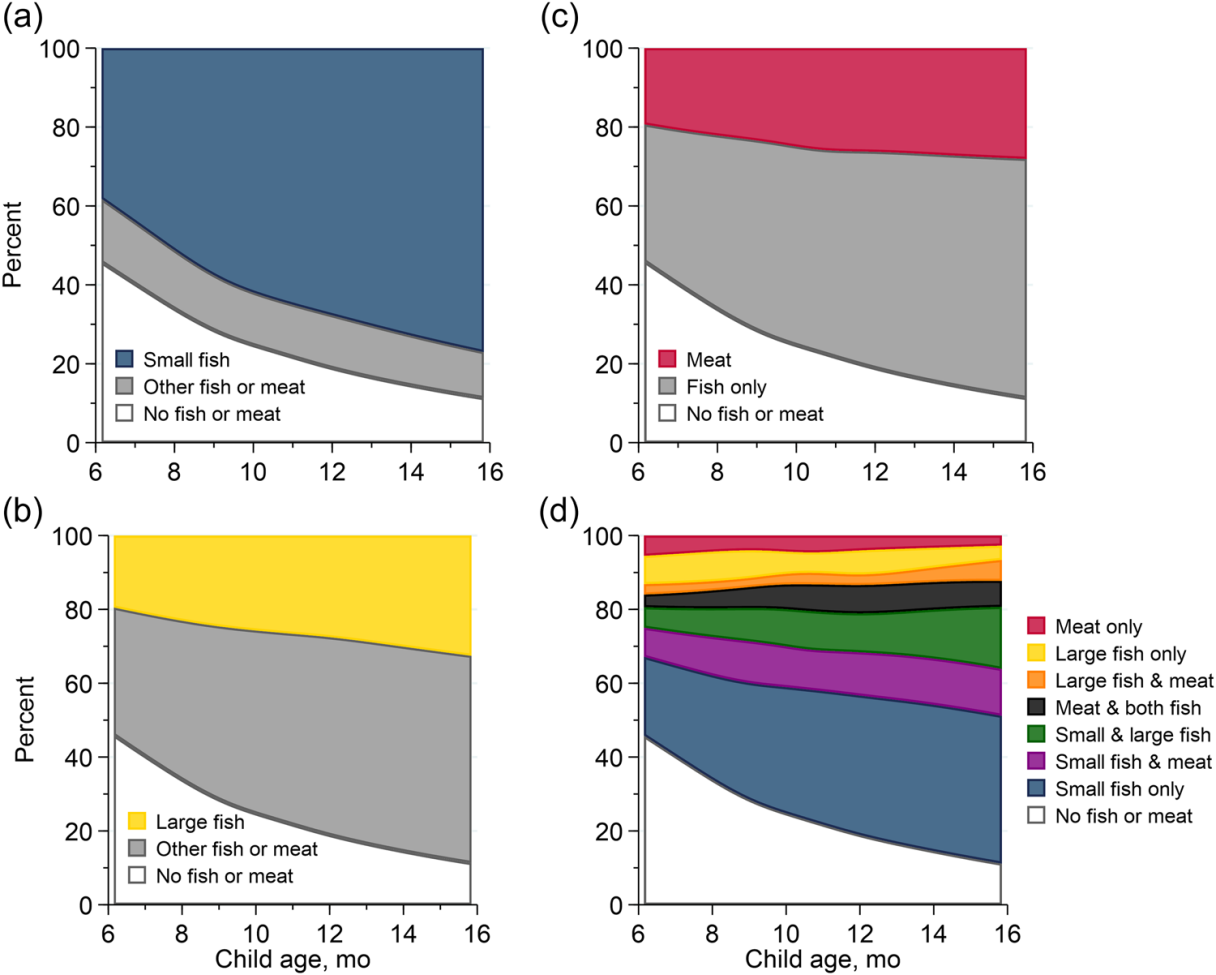
Weekly flesh food consumption among children aged 6–15 months in Mangochi, Malawi, 2018–2019.

Over the 6‐month study period, children consumed small fish on a median of 24% (25th and 75th percentile: 14, 34) of days, large fish on 5% (2, 12) of days and meat on 4% (2, 9) of days (Supporting Information: Figure 2). The proportion of children consuming any fish or meat in the past 7 days increased with child age: 69% at 6–9 months, 78% at 9–12 months and 84% at 12–15 months (Figure 2). Similarly, small fish consumption on one or more days within the past week increased with child age: 55% at 6–9 months, 64% at 9–12 months and 70% at 12–15 months (Figure 2a). Flesh food consumption was infrequently reported in 24‐h dietary recalls at enrolment (6–9 months of age; 6%) but increased by midline (9–12 months of age; 36%) and endline (12–15 months of age; 57%) (Table 1). At endline, children consumed mean usual intakes of 1.2 g/day meat, 1.2 g/day large fish and 4.9 g/day small fish (Table 1).

**Table 1:** Intake of flesh foods from 24‐h dietary recalls of young children in Mangochi, Malawi: Mazira Project 2018–2019.^a^

Flesh food	Consumers,^b^ *n* (%)			Usual intake (g/day)^c^		
	Enrolment	Midline	Endline	Enrolment^d^	Midline	Endline
Small fish	25 (4)	144 (24)	239 (40)	‐‐	1.8 (0.6, 4.5)	4.9 (2.3, 8.7)
Large fish	4 (1)	38 (6)	74 (12)	‐‐	0.5 (0.2, 1.3)	1.2 (0.5, 2.6)
Meat	10 (2)	39 (7)	54 (9)	‐‐	0.7 (0.3, 1.6)	1.2 (0.6, 2.1)

^a^ Children were 6–9 months old at enrolment, 9–12 months old at midline and 12–15 months old at endline visits.

^b^ Consumers are the number and percentage of children with reported intakes of small fish, large fish or meat in the past 24‐h among all children who completed a 24‐h dietary recall at the study visit.

^c^ Usual intake reported as median (p25, p75) using the *DISTRIB* macro from the National Cancer Institute (2022).

^d^ Missing values (‐‐) due to failed convergence of the two‐part, correlated model due to too few consumers.

### 3.3 Associations between usual intake from ASF screeners and iron indices

Greater frequency of small fish intake was associated with small improvements in iron status (Table 2). Over 6 months, each increment of 10% of days of small fish consumption (analogous to one additional consumption day per every 10 days) was associated with a relative 2% lower sTfR concentration, such as a 0.2 mg/L decrease from the mean sTfR of 11.4 mg/L. Similarly, there was a positive association between plasma ferritin and the percent of weeks during which any flesh foods were consumed. For each 10% increment in percent of weeks of any flesh food intake (corresponding to a minimum frequency of once in 2.3 weeks), ferritin concentrations were 3% higher. Over the 3 months preceding the endline visit, percent of days with small fish intake was not associated with any iron indices, but the percent of weeks with any flesh food intake was associated with lower sTfR.

**Table 2:** Association between percent of days fish and meat were consumed and iron and anaemia among young children in Mangochi, Malawi: Mazira Project, 2018–2019.^a^

	Ferritin (µg/L)	sTfR (mg/L)	Hgb (g/dL)	Anaemia (%)	ID (%)	IDA (%)
	Geometric mean (p25, p75)	Geometric mean (p25, p75)	Geometric mean (p25, p75)			
Inflammation‐adjusted value at endline (12–15 months old)^b^	7.1 (4.1, 11.4)	11.4 (8.6, 14.7)	11.0 (10.3, 12.0)	43	89	40
	GMR (95% CI)	GMR (95% CI)	GMR (95% CI)	PR (95% CI)	PR (95% CI)	PR (95% CI)
Intake over past 6 months^c^^,^ d						
Small fish, per 10% of days	1.03 (0.98, 1.07)	**0.98 (0.96, 1.00)**	1.01 (1.00, 1.01)	0.95 (0.88, 1.02)	1.00 (0.98, 1.02)	0.95 (0.87, 1.03)
Large fish, per 10% of days	1.05 (0.98, 1.12)	0.99 (0.96, 1.02)	0.99 (0.98, 1.00)	**1.09 (1.01, 1.19)**	**0.96 (0.93, 1.00)**	1.04 (0.95, 1.15)
Meat, per 10% of days	1.04 (0.94, 1.16)	0.98 (0.93, 1.03)	1.01 (0.99, 1.02)	1.07 (0.91, 1.25)	1.00 (0.96, 1.05)	1.10 (0.93, 1.31)
Any flesh food, per 10% of weeks	**1.03 (1.00, 1.06)**	0.99 (0.98, 1.00)	1.00 (1.00, 1.01)	1.00 (0.95, 1.04)	0.99 (0.98, 1.01)	0.99 (0.94, 1.04)
Intake over past 3 months^c^^,^ e						
Small fish, per 10% of days	1.02 (0.99, 1.06)	0.99 (0.97, 1.00)	1.00 (1.00, 1.01)	0.96 (0.90, 1.02)	1.00 (0.98, 1.01)	0.96 (0.90, 1.03)
Large fish, per 10% of days	1.02 (0.97, 1.08)	0.99 (0.97, 1.02)	1.00 (0.99, 1.01)	1.04 (0.96, 1.12)	**0.97 (0.94, 1.00)**	1.02 (0.94, 1.10)
Meat, per 10% of days	1.02 (0.94, 1.12)	0.98 (0.94, 1.02)	1.01 (0.99, 1.02)	1.06 (0.93, 1.21)	1.02 (0.98, 1.06)	1.10 (0.96, 1.26)
Any flesh food, per 10% of weeks	1.02 (1.00, 1.05)	**0.99 (0.97, 1.00)**	1.00 (1.00, 1.01)	0.99 (0.95, 1.04)	1.00 (0.98, 1.01)	0.99 (0.94, 1.03)

*Note*: Bold values show GMRs or PRs with significance of *p* < 0.05.

Abbreviations: GMR, geometric mean ratio; Hgb, haemoglobin; ID, iron deficiency; IDA, iron deficiency anaemia; PR, prevalence ratio; sTfR, soluble transferrin receptor.

^a^ Ferritin (*n* = 575), sTfR (*n* = 575), Hgb (*n* = 585); anaemia (*n* = 585); ID (*n* = 575); IDA (*n* = 568). All models adjusted for malaria, month of assessment, child sex, child age, child illness and baseline measures. Ferritin, sTfR, ID and IDA models included adjustment for inflammation. Health centre, maternal education, number of children in the household under 5 years and minutes between blood draw and aliquot completion were included when *p* < 0.1.

^b^ Ferritin, sTfR, ID and IDA models adjusted for inflammation using the Biomarkers Reflecting Inflammation and Nutritional Determinants of Anaemia (BRINDA) approach (Namaste et al., 2017; Rohner et al., 2017).

^c^ Dietary exposures for small fish, large fish and meat were calculated as percent of days among available ASF screeners to include participants with varying numbers of missing ASF screeners. Any flesh food intake was totalled by week rather than days because the ASF screener asked respondents for the number of consumption days within the past week, rather than asking respondents to specify which days foods were consumed.

^d^ 10% of days refers to increments of approximately 16 days and 10% of weeks refers to increments of approximately 2.3 weeks.

^e^ 10% of days refers to increments of approximately 9 days and 10% of weeks refers to increments of approximately 1.3 weeks.

Greater frequency of large fish intake over 6 months was associated with higher anaemia prevalence and lower iron deficiency prevalence but not mean haemoglobin, ferritin or sTfR (Table 2). Over 6 months, each 10% of days increment in large fish intake was associated with a relative 9% higher prevalence of anaemia and 4% lower prevalence of iron deficiency. Over the past 3 months, frequency of large fish intake was associated with lower iron deficiency but not anaemia. Frequency of meat consumption over 6 months and the past 3 months was not associated with any iron or anaemia indices.

### 3.4 Associations between usual intake from 24‐h dietary recalls and iron indices

At endline, usual intake of flesh foods as measured in grams of the food reported in 24‐h recalls was not associated with any iron or anaemia indices (Table 3). While models for small fish had a high rate of convergence (≥98%), large fish and meat models had moderate convergence (approximately 75%), primarily due to the high number of zero consumption days. At midline, small fish models converged with ≥96% bootstrap iterations and were not associated with iron or anaemia indices (Table 3). However, models for large fish and meat at 9–12 months were excluded from analysis due to poor convergence (approximately 50%–60%) among bootstrap iterations.

**Table 3:** Association between predicted usual intake of flesh foods from 24‐h dietary recalls with iron and anaemia at 12–15 months of age in the Mazira Project, Malawi, 2018–2019.^a^

	Ferritin (µg/L)	sTfR (mg/L)	Hgb (g/dL)	Anaemia (%)	ID (%)	IDA (%)
	GMR (95% CI)	GMR (95% CI)	GMR (95% CI)	PR (95% CI)	PR (95% CI)	PR (95% CI)
Endline (12–15 months)^b^						
Small fish, per 1 g	1.02 (0.97, 1.07)	1.00 (0.98, 1.02)	1.00 (0.99, 1.01)	1.01 (0.95, 1.08)	1.01 (0.99, 1.03)	1.04 (0.97, 1.11)
Large fish, per 1 g	1.10 (0.93, 1.30)	0.99 (0.90, 1.10)	0.99 (0.95, 1.02)	1.06 (0.80, 1.40)	0.91 (0.73, 1.14)	1.03 (0.67, 1.57)
Meat, per 1 g	1.09 (0.72, 1.66)	0.90 (0.67, 1.21)	1.00 (0.91, 1.10)	0.99 (0.46, 2.14)	1.18 (0.92, 1.51)	1.01 (0.26, 3.95)
Midline (9–12 months)^c^						
Small fish, per 1 g	0.99 (0.86, 1.15)	0.96 (0.89, 1.03)	1.01 (0.99, 1.04)	0.9 (0.68, 1.18)	1.02 (0.95, 1.09)	0.95 (0.73, 1.23)

Abbreviations: GMR, geometric mean ratio; Hgb, haemoglobin; ID, iron deficiency; IDA, iron deficiency anaemia; sTfR, soluble transferrin receptor; PR, prevalence ratio.

^a^ Ferritin (*n* = 575); sTfR (*n* = 575); Hgb (*n* = 585); anaemia (*n* = 585); ID (*n* = 575); IDA (*n* = 568). All results adjusted for malaria, month of assessment, child sex, child age, child illness and baseline measures. Ferritin, sTfR, ID and IDA models adjusted for inflammation using the Biomarkers Reflecting Inflammation and Nutritional Determinants of Anaemia (BRINDA) approach (Namaste et al., 2017; Rohner et al., 2017). Health centre, maternal education, the number of children in the household under 5 years and minutes between blood draw and aliquot completion were included when *p* < 0.1.

^b^ A 1 g higher usual intake from the median corresponds to the following percentiles: 57th for small fish, 70th for large fish and 76th for meat.

^c^ A 1 g higher usual intake from the median corresponds to the 62nd percentile for small fish.

### 3.5 Sensitivity and post hoc analyses

In sensitivity analyses examining the influence of missing ASF screeners on our models, we imputed or dropped observations and compared them to models of intake over 6 months (Supporting Information: Table 4). Imputing observations from missing ASF screeners did not substantially impact the point estimates (≤1 percentage‐point difference) but did reduce the statistical significance of the association between days of small fish intake and sTfR, days of large fish intake and anaemia and weeks of any flesh food intake and ferritin. Dropping observations from 13 children missing more than 50% of ASF screeners did not impact the point estimates or significance of associations apart from the large fish intake and anaemia model.

We further explored the associations of large fish with anaemia and iron deficiency in a series of post hoc analyses. Frequent consumers of large fish tended to be more likely to consume small fish as well. Additionally, they were more likely to own chickens, complete more weekly ASF screeners, have malaria at enrolment and live in the Lungwena catchment area. Since households in the two catchment areas (Lungwena and Malindi) differed in composition of ethnicity, religion and level of educational attainment, catchment area may serve as a proxy variable for a combination of measured and unmeasured characteristics. Large fish consumption was no longer associated with iron deficiency after adjusting for malaria at enrolment, whereas neither the point estimate nor the significance of association for the large fish and anaemia model were impacted by addition of demographic variables as covariates. However, the association between large fish and anaemia was no longer statistically significant after adjusting for CRP at endline.

## 4 DISCUSSION

In this sample of young children from fishing communities along the southeastern shore of Lake Malawi, small fish were the most consumed flesh food and the primary dietary source of estimated absorbable iron at endline (12–15 months of age). More frequent small fish consumption was associated with moderately improved iron status. Large fish showed less potential to improve iron status than small fish because it was associated with iron deficiency but not ferritin or sTfR, and meat was not associated with any iron or anaemia indices. Though frequency of fish and meat consumption increased with child age, average usual intakes remained small (1.2 g/day meat, 1.2 g/day large fish, and 4.9 g/day small fish at endline). Small portion sizes may explain the weak association between frequency of consumption and iron status as well as the lack of association between usual intakes and iron status.

Our finding that small fish are associated with moderate improvements to iron status of young children is consistent with the nutrient profile of small, Malawian fish (MAFOODS, 2019) as well as ecologic and economic factors favoring consumption of small fish. Small fish are typically consumed whole, providing a good dietary source of bioavailable haem iron from the fish's head, flesh, and organs. As a nutrient‐dense food, small fish are especially important for optimising nutrient intakes of young children in low‐ and middle‐income countries (Beal & Ortenzi, 2022; Bogard et al., 2015; Byrd, Pincus, et al., 2021; Byrd et al., 2022; Gibson et al., 2003; Parlesak et al., 2014; White et al., 2021). Previous studies have indicated that small fish harvested at maturity can be part of a nutritious, locally sourced, environmentally sustainable, and affordable diet for Malawian children (Global Alliance for Improved Nutrition (GAIN) & United Nations Children's Fund (UNICEF), 2021; Lutter et al., 2021; National Statistical Office NSO Malawi, & ICF, 2017; O'Meara et al., 2021; Simmance et al., 2021). Small fish are more widely available in open‐air markets than large fish. In 2019, over 60% of fish caught by Malawian capture fisheries were small freshwater lake sardines like *usipa* (Food and Agriculture Organization, 2021). Although large fish may be accessible to study households given their proximity to the southeast shore of Lake Malawi and employment within the fishing industry, this region is socioeconomically disadvantaged and there are financial incentives to sell large fish and consume small fish instead. In July 2018, one *chambo* (tilapia) fish (approximately 359 g) could be purchased locally for nearly a day's wages (1500 kwacha or $2.00 USD), whereas a small stack of *usipa* or *kambuzi* small fishes (approximately 102 g) cost 200 kwacha ($0.30 USD) (Bennett et al., 2022; Chikowi et al., 2021). In Malawi, household wealth is one of the primary determinants of both quantity and frequency of fish consumption (Chikowi et al., 2021), and households with low socioeconomic status may reserve more expensive large fish—especially *chambo*—for celebrations and other special occasions. However, market availability and affordability for some households does not guarantee children's consumption of animal source foods (Creed‐Kanashiro et al., 2018; Haileselassie et al., 2020; Pachón et al., 2007).

Our study found that small fish consumption increased with the age of the child, suggesting that infant and young child feeding practices may be influenced by perception of developmentally appropriate foods or intrahousehold allocation. In the present study, caregivers completing 24‐h dietary recalls frequently reported that only the sauce or broth from mixed dishes containing fish were provided to children (Caswell et al., 2021). Their rationale for withholding fish flesh to study infants and young children may be represented by statements from women in focus groups who prepared fish dishes for collection of weighed recipes: children may choke or not be able to chew meat because they lack teeth and have not learned to pick out bones or are otherwise too young to eat fish that should be given to other (older) members of the household instead. Similarly, in Senegal children without teeth may not be offered meat, and in Peru and Bangladesh caregivers of young children may refrain from offering fish because of concerns with choking or time required to pick out the bones (Pachón et al., 2007; Thorne‐Lyman et al., 2017). Thus, to increase fish intake of infants, whole small fish containing the head, bones, and organs may be ground into a powder or paste for mixing into complementary foods (Bogard et al., 2015; Byrd, Thilsted, et al., 2021; Kawarazuka & Béné, 2011). Additionally, foods offered to infants may be cooked separate from household foods, prepared in‐between meals of other household members, or guided by cultural norms of foods appropriate for the role or status of each household member. For example, infants in fish‐farming households in rural Bangladesh were more likely to consume eggs, dairy, and organ meats and less likely to consume fish than their mothers (Thorne‐Lyman et al., 2017), and in rural Malawi, choice meats and large fish may be preferentially given to older children or adults instead of infants, who may predominantly receive broth only.

To date, no studies to our knowledge have examined the effect of providing dried fish powder alone or fish on iron status of infants in low‐ and middle‐income countries in comparison to an unenriched or nonintervention control (Byrd et al., 2022). However, three trials found plasma ferritin, sTfR, percent transferrin saturation, and haemoglobin did not differ between infants and young children randomised to receive porridges commercially fortified with micronutrients and fish powder (Konyole et al., 2019; Lartey et al., 1999; Skau et al., 2015). Among school age children in Ghana, provision of cowpeas fortified with fish powder for 6mo resulted in higher haemoglobin but similar ferritin to children receiving nonfortified cowpeas (Egbi et al., 2015). Among 3–7 year old Bangladeshi children, providing an iron‐rich mola fish curry dish six days per week for nine weeks improved mean sTfR but not ferritin or haemoglobin concentrations compared to children in the less iron‐rich rui fish control group (Anderson et al., 2016). These studies establish some precedent of the potential for fish consumption to improve iron and anaemia status of young children in low‐and‐middle income countries, although the reported effects have been relatively small and inconsistent.

In our analysis, large fish and meat were either not associated with iron or the associations were attenuated by controlling for additional covariates. This may be related to both variability in iron density of foods grouped by category and infrequent consumption of large fish and meat. Large fish consumption may not contribute to iron status as much as small fish because the fillets from large fish are less iron‐dense than whole small fish, which includes more haem iron from organs. However, nutrient analyses for freshwater fish species are limited, and nutrient composition tables indicate considerable variation between species (Byrd, Thilsted, et al., 2021; MAFOODS, 2019). In this study, meat consumption was predominantly chicken, which has less haem and lower iron density than red meats like beef and goat meat. Results from a cluster‐randomised efficacy trial conducted in multiple low‐and‐middle income countries indicated that infants with greater intake of red meat (30–45 g/day beef) can maintain their iron status through 18 months of age (Krebs et al., 2012), however, we are aware of no similar studies of chicken meat. The combination of infrequent meat consumption, small portions, and relatively lower iron content of chicken may explain why meat was not associated with iron indices in our study population. Since large fish consumption had a low prevalence and was not associated with ferritin, sTfR, or haemoglobin, the associations of large fish with lower iron deficiency and higher anaemia may be spurious or influenced by other explanatory factors not included as covariates, such as malaria at enrolment or CRP at endline.

Though frequency of flesh food consumption over several months showed some relationships with iron and anaemia, usual intake of flesh foods reported through 24‐h dietary recalls at endline was not associated with iron or anaemia. The differences in findings may be explained by the definitions and methods of quantifying exposure. First, classification of small versus large fish may have differed between the ASF screener and 24‐h dietary recall. In the ASF screener, respondents determined the size classification of the fish, which may be based on fishing industry standards for length, such as <25 cm (Simmance et al., 2021). In the 24‐h dietary recall, we classified small fish as species that were likely to be consumed whole, presuming these fish to have higher iron content. Additionally, interviewers conducting 24‐h dietary recalls probed for details on how mixed dishes were consumed, specifically differentiating between intake of broth only versus fish flesh when caregivers reported fish soup, whereas ASF screener respondents may have counted the broth of fish soup towards days of fish consumption. Second, the 24‐h dietary recalls capture recent short‐term exposure, which may differ from long‐term exposures of usual intake. Accordingly, nutrition biomarkers reflecting iron stores may be more sensitive to measures of cumulative rather than short‐term dietary intake. Third, applying the NCI method for assessing usual intake of episodically consumed foods presented several challenges in this population of young children. The data set contained many values of 0 g intake for fish and meat, which limited the convergence of two‐part correlated models at enrolment and midline. For analytical models performed on the log‐transformed scale, zero intakes were replaced with one‐quarter of the minimum quantity reported in the 24‐h recalls. However, replacing 60%–91% of values with any small number can bias the estimate of mean usual intake as well as artificially reduce the variability of measured dietary intakes, which may additionally bias the relationship with nutrition biomarkers towards the null. Performing sensitivity analyses for various imputation methods would be ideal; however, this is not always feasible due to the long total computing time required to run the full set of bootstrapped analytical models. Since fish and meat are episodically consumed foods, we recommend quantification of dietary intakes of young children by frequency over time for comparison to nutritional biomarkers and use of 24‐h dietary recalls for supporting information on portion sizes.

This analysis presented with several limitations concerning the analysis and generalisability of findings. Some selection bias may be present because approximately 13% of children had incomplete iron measurements, most of whom withdrew early in the study period. Consumption of any flesh foods could only be aggregated by the percent of weeks rather than percent of days, which would improve the ease of comparison across analytical models as well as with infant and young child feeding recommendations for animal‐source foods. Though dietary iron intake from flesh foods may have a more direct relationship with iron biomarkers, we used grams of flesh food intake because food composition data and iron content are limited for many freshwater fish species (Byrd, Thilsted, et al., 2021). If red meat or organs were consumed by a greater proportion of the population, it would have been advantageous to analyse these separately from less haem iron‐rich foods like chicken. Conducting additional repeat dietary recalls would improve the precision for estimated usual intake of infrequently consumed large fish and meat and their relationship with iron and anaemia biomarkers. Our study did not measure other sources of anaemia such as hemoglobinopathies and intestinal parasite infection, which does not allow us to distinguish between nutritional anaemias and those due to other causes; nor does it allow us to adjust for potential confounding due to these other factors. Though iron biomarkers were adjusted for inflammation, the high burden of inflammation in the study population may have inhibited the impact of dietary iron intake of flesh foods and biomarkers of iron status and anaemia. This is because the inflammatory response can not only sequester iron into tissue stores but also impair absorption of dietary iron. Therefore, future research should aim to control infections like malaria and enteric diseases and inflammation before evaluating relationships between dietary intakes and iron deficiency or anaemia.

Some of the strengths of this analysis include assessing usual intake by different data sources, conducting multiple sensitivity analyses and examining the potential for locally available foods to alleviate iron deficiency. The 24‐h dietary recall was conducted in such a way as to disaggregate ingredients of mixed dishes before quantifying intake of animal‐source foods. This is particularly important for infants and young children who may only be fed some ingredients of a family meal. Using both ASF screeners and 24‐h dietary recalls captures different periods of exposure and provides a complementary and more comprehensive assessment of usual intake because they are prone to different biases and sources of measurement error (Kirkpatrick et al., 2022). Quantitative assessment of usual intake was strengthened by applying the NCI method to adjust for within‐person variation in daily intake. Numerous sensitivity analyses were conducted on the ASF screener models and provided consensus on the magnitude and significance of associations as compared to principal models. Importantly, this analysis contributed towards filling a literature gap concerning the relationship between iron‐specific biomarkers and iron‐rich flesh foods in young children. Contextually, this is significant because young Malawian children enroled in this study have a high burden of iron deficiency and access to fish as an iron‐rich food source.

## 5 CONCLUSION

The potential for small fish to improve dietary adequacy of iron in infants and young children is gaining interest (Beal & Ortenzi, 2022; White et al., 2021); yet, few studies have examined the relationship between fish consumption and iron indices or other nutritional biomarkers (Byrd et al., 2022; Kawarazuka & Béné, 2011). This study is one of few to examine the relationship of fish and meat with iron biomarkers among children in low‐and‐middle income countries. Our findings suggest that even a small increase in small fish consumption, such as 2 days per month, could marginally improve iron status of infants and young children; however, these findings may be specific to our study context in which children lived in Malawian fishing communities, ate small portion sizes of flesh foods and had high burdens of iron deficiency and inflammation (Caswell et al., 2021; Lutter et al., 2021; Werner et al., 2022). If the average intake of small fish doubled, such that children consumed small fish every other day, we would expect ferritin to increase by 0.5 µg/L and sTfR to decrease by 0.5 mg/L; however, the proportion of iron deficient children would remain similar (89%), given the severity of iron deficiency in this context. Additionally, the high burden of inflammation may limit absorption of dietary iron, mobilisation of iron stores and response to dietary intake interventions and also contribute to the burden of nonnutritional anaemias in this region.

Further research is needed to examine the relationship of fish and meat intake with iron biomarkers in contexts with less inflammation and larger portion sizes, as well as in comparison to nonintervention or control groups provided with unenriched foods (Byrd et al., 2022; Konyole et al., 2019; Lartey et al., 1999; Lin et al., 2008; Skau et al., 2015). Additionally, future research concerning infant and young child feeding interventions should consider barriers to consumption of fish, meat and other animal‐source foods among young children beyond physical access and availability, such as affordability and the unique beliefs and practices surrounding infant and young child feeding. Barriers to consumption among young children likely differ from those of other household members. Strategies to increase fish consumption among children may include preserving small‐scale fisheries, supporting the value chain to distribute fish to inland communities and adopting market‐based approaches to increase household purchasing power, reduce market price and develop innovative products, such as nutritional supplements or ready‐to‐use foods containing fish powder (Byrd et al., 2022; Konyole et al., 2019; Lartey et al., 1999; Lin et al., 2008; Skau et al., 2015). Alternatively, community health workers could provide instruction to caregivers on texture‐ and age‐appropriate ways to prepare fish for infants (Gibson et al., 2003) and the recommended frequency and portions of animal‐source foods to meet infants' nutritional needs. Lastly, dietary strategies to reduce the burden of iron deficiency in young children may vary by context and involve a variety of approaches, such as promoting flesh food intake (Chouraqui, 2022), enhancing the bioavailability of plant‐based iron sources (Manary et al., 2002) and provision of iron‐containing nutritional supplements to infants and young children (Suchdev et al., 2020; Wessells et al., 2021).

## AUTHOR CONTRIBUTIONS

*Designed research*: Christine P. Stewart, Bess L. Caswell, Lora L. Iannotti, Kenneth M. Maleta, E. Rochelle Werner. *Conducted research*: Christine P. Stewart, Kenneth M. Maleta, Bess L. Caswell, Charles D. Arnold, E. Rochelle Werner. *Analysed data or performed statistical analysis*: E. Rochelle Werner, Charles D. Arnold. *Wrote paper*: E. Rochelle Werner, Christine P. Stewart. *Primary responsibility for final content*: E. Rochelle Werner, Christine P. Stewart. All authors have read and approved the manuscript for submission.

## CONFLICT OF INTEREST STATEMENT

The authors declare no conflict of interest.

## Supporting information

Supporting information.

## Data Availability

The data that support the findings of this study are available from the corresponding author upon reasonable request.
