# Supplementary material for: Associations of fish and meat intake with iron and anaemia in Malawian children
Source: Matern Child Nutr. 2024 Jan 12;20(2):e13622. doi: 10.1111/mcn.13622 (PMC10981481; doi:10.1111/mcn.13622)
Supplement: Supplementary file 1 — Supporting information. [file MCN-20-e13622-s001.pdf]

Associations of Fish and Meat Intake with Iron and Anaemia in Malawian Children

E. Rochelle Werner[1,2], Charles D. Arnold[1], Bess L. Caswell[1,3], Lora L. Iannotti[4], Kenneth M. Maleta[5], Christine P. Stewart[1]*

[1]Institute for Global Nutrition, University of California, Davis, Davis, CA, USA

[2]Department of Global Health, Rollins School of Public Health, Emory University, Atlanta, GA, USA

[3]Western Human Nutrition Research Center, U.S. Department of Agriculture, Davis, CA, USA

[4]E3 Nutrition Lab, Washington University in St. Louis, St. Louis, MO, USA

[5]School of Global and Public Health, Kamuzu University of Health Sciences, Blantyre, Malawi

How to cite this article: Werner, E.R., Arnold, C.D., Caswell, B.L., Iannotti, L.L., Maleta, K.M., & Stewart, C.P. (2023). Associations of fish and meat intake with iron and anaemia in Malawian children. *Maternal & Child Nutrition*, e13622. https://doi.org/10.1111/mcn.13622

Supplementary Table 1: Adjustment factors for the bioavailability of dietary iron for children in the Mazira Project, Malawi, 2018-2019

Category	Factor	Reference
Grains	1.8%	1.8% maize (Hurrell, RF et al. *Am J Clin Nutr*. 2003;77:1213-9)
Legumes/nuts	1.6%	1.6% peanuts (Petry et al. *J. Nutr*. 2010;140:1977-1982)
Vit A-rich F&V		
Leafy greens	1.2%	1.2% broccoli w/ 2g phytate (Gillooly, M et al. *British J of Nutr*. 1984;5:37-46)
Dark orange F&V	10%	10% from moderate iron bioavailable infant diet (IOM 2001)
Other F&V	10%	10% from moderate iron bioavailable infant diet (IOM 2001)
Small fish[1]	22%	~30% (up to 40%) heme iron in fish (Cook, JD & Monsen, ER. *Am J Clin Nutr*. 1976;29:859-67)
Large fish[1]	22%	~30% (up to 40%) heme iron in fish (Cook, JD & Monsen, ER. *Am J Clin Nutr*. 1976;29:859-67)
Meat[1]	22%	~30% heme iron in white meat (Hurrell, R. *The mineral fortification of foods*. Leatherhead: Leatherhead International Ltd; 1999. pp. 54–93. [As cited in Lynch, S et al. *J Nutr*. 2018; 148:1001S–1067S])
Organs[1]	30%	~70% heme iron in red meat (Hurrell R. *The mineral fortification of foods*. Leatherhead: Leatherhead International Ltd; 1999. pp. 54–93. [As cited in Lynch, S et al. *J Nutr*. 2018; 148:1001S–1067S])
Milk	10%	10% from moderate iron bioavailable infant diet (IOM 2001)
Eggs	3.7%	3.7% hen eggs (Callender, ST et al. *British J of Haem*. 1970;19:657-65)
Other	10%	10% from moderate iron bioavailable infant diet (IOM 2001)

F&V = fruits and vegetables

[1]Assumes 35% absorption of heme iron (Hurrell, R. *Am J Clin Nutr*. 2010; 91(suppl):1461S–7S.) and 16.8% absorption of non-heme iron from flesh food-rich diets (IOM 2001)

Supplementary Table 2: Enrollment characteristics of children in the Mazira Project, Malawi, 2018-2019[1]

Characteristic	*n*	value
Child		
Child age, mo	660	7.4 ± 1.2
Female, %	660	48
Breastfeeding, %	659	100
Foods consumed in past 24hrs		
Small fish, %	659	4
Large fish, %	659	1
Meat, %	659	2
Organs, %	659	0
Dairy, %	659	7
Eggs, %	659	4
Legumes and beans, %	659	36
Grains and cereals, %	659	99
Vitamin A-rich fruits and vegetables, %	659	25
Other fruits and vegetables, %	659	52
Inflammation (CRP >5mg/L or AGP >1g/L)	525	62
Positive malaria test (RDT), %	595	13
Hemoglobin, g/dL	582	10.4 (9.5, 11.5)
Plasma ferritin, µg/L	525	25.9 (13.2, 50.4)
Plasma sTfR, mg/L	525	11.4 (9.0, 15.4)
Anemia (hemoglobin <11g/dL), %	582	61
Any iron deficiency[4], %	525	85
Iron deficiency anemia, %	524	55
Inflammation-adjusted[5]		
Plasma ferritin, µg/L	525	14.1 (7.9, 25.5)
Plasma sTfR, mg/L	525	9.7 (7.9, 13.1)
Any iron deficiency[4], %	525	77
Iron deficiency anemia, %	524	52
Maternal		
Maternal age, y	654	26.0 ± 6.7
Maternal primary education[2], %	660	20
Maternal literacy, %	643	46
Household		
Number of children under 5 y	638	1.7 ± 0.8
Number of household members	641	5.9 ± 2.7
Moderate or severe food insecurity[3], %	660	78

[1]Values are %, mean ± SD, or median (P25, P75). AGP = α-1-acid glycoprotein; CRP = c-reactive protein; RDT = rapid diagnostic test; sTfR = soluble transferrin receptor
[2]Percent completed primary or greater
[3]Food insecurity assessed using Household Food Insecurity Access Scale (Coates et al., 2007)
[4]Ferritin <12µg/L and/or sTfR >8.3mg/L
[5]Inflammation-adjusted using the BRINDA (Biomarkers Reflecting Inflammation and Nutritional Determinants of Anemia) approach (Namaste et al., 2017; Rohner et al., 2017)

Supplementary Table 3: Endline characteristics of children included in the fish and meat analysis of the Mazira Project, Malawi, 2018-2019[1]

Child characteristics	*n*	value
Child age, mo	585	13.3 ± 1.2
Female, %	585	48
Breastfeeding, %	585	99
Foods consumed in past 24hrs		
Small fish, %	580	41
Large fish, %	580	13
Meat, %	580	9
Organs, %	580	1
Dairy, %	580	19
Eggs, %	580	38
Legumes and beans, %	580	46
Grains and cereals, %	580	99
Vitamin A-rich fruits and vegetables, %	580	40
Other fruits and vegetables, %	580	92
Inflammation (CRP >5mg/L or AGP >1g/L)	575	51
Positive malaria test (RDT), %	585	6
Hemoglobin, g/dL	585	11.1 (10.3, 12.0)
Plasma ferritin, µg/L	575	11.0 (6.3, 21.2)
Plasma sTfR, mg/L	575	11.9 (9.0, 15.9)
Anemia (hemoglobin <11g/dL), %	585	43
Any iron deficiency[4], %	575	87
Iron deficiency anemia, %	575	40
Inflammation-adjusted[5]		
Plasma ferritin, µg/L	575	6.0 (3.8, 10.8)
Plasma sTfR, mg/L	575	11.0 (8.6, 14.7)
Any iron deficiency[4], %	575	89
Iron deficiency anemia, %	575	40

[1]Values are %, mean ± SD, or median (P25, P75). AGP = α-1-acid glycoprotein; CRP = c-reactive protein; RDT = rapid diagnostic test; sTfR = soluble transferrin receptor. Children included in analysis were required to have hemoglobin assessed at the 6-month follow-up visit. Maternal and household characteristics were not reassessed at endline.

[2]Percent completed primary or greater

[3]Food insecurity assessed using Household Food Insecurity Access Scale

[4]Ferritin <12µg/L and/or sTfR >8.3mg/L

[5]Inflammation-adjusted using the BRINDA (Biomarkers Reflecting Inflammation and Nutritional Determinants of Anemia) approach (Namaste et al., 2017; Rohner et al., 2017)

Supplementary Table 4: Sensitivity analyses for imputed and dropped observations conducted for the association between percent of days of flesh food intake over 6mo and iron and anemia at 12-15mo of age in the Mazira Project, Malawi, 2018-2019[1]

	Ferritin, μg/L GMR (95%CI)	sTfR, mg/L GMR (95%CI)	Hgb, g/dL GMR (95%CI)	Anemia, % PR (95%CI)	ID, % PR (95%CI)	IDA, % PR (95%CI)
Imputed observations[2]						
Small fish, per 10% of days	1.02 (0.98, 1.07)	0.98 (0.96, 1.00)	1.01 (1.00, 1.01)	0.95 (0.89, 1.02)	1.00 (0.98, 1.02)	0.95 (0.88, 1.03)
Large fish, per 10% of days	1.05 (0.98, 1.13)	0.99 (0.96, 1.02)	0.99 (0.98, 1.00)	1.08 (1.00, 1.18)	**0.96 (0.92, 1.00)**	1.03 (0.94, 1.14)
Meat, per 10% of days	1.05 (0.95, 1.16)	0.98 (0.93, 1.02)	1.01 (0.99, 1.02)	1.06 (0.91, 1.24)	1.00 (0.96, 1.05)	1.10 (0.92, 1.30)
Any flesh food, per 10% of weeks	1.02 (0.96, 1.08)	1.00 (0.98, 1.03)	1.00 (0.99, 1.01)	0.99 (0.91, 1.08)	1.01 (0.98, 1.05)	0.93 (0.84, 1.02)
Dropped observations[3]						
Small fish, per 10% of days	1.03 (0.98, 1.07)	**0.98 (0.96, 1.00)**	1.01 (1.00, 1.01)	0.94 (0.88, 1.01)	1.00 (0.98, 1.02)	0.94 (0.86, 1.02)
Large fish, per 10% of days	1.06 (0.99, 1.13)	0.99 (0.96, 1.02)	0.99 (0.98, 1.00)	1.09 (1.00, 1.18)	**0.96 (0.92, 0.99)**	1.04 (0.94, 1.15)
Meat, per 10% of days	1.04 (0.94, 1.16)	0.98 (0.93, 1.03)	1.01 (0.99, 1.02)	1.09 (0.94, 1.27)	1.01 (0.96, 1.06)	1.14 (0.96, 1.34)
Any flesh food, per 10% of weeks	**1.03 (1.00, 1.06)**	0.99 (0.98, 1.00)	1.00 (1.00, 1.01)	0.99 (0.95, 1.04)	0.99 (0.98, 1.01)	0.98 (0.94, 1.03)

GMR = geometric mean ratio; Hgb = hemoglobin; ID = iron deficiency; IDA = iron deficiency anemia; sTfR = soluble transferrin receptor; PR = prevalence ratio

[1]Ferritin (n=575), sTfR (n=575), Hgb (n=585); anemia (n=585); ID (n=575); IDA (minimally-inflammation adjusted: n=575; fully adjusted: n=568). All models adjusted for malaria, month of assessment, child sex, child age, child illness, and baseline measures. Ferritin, sTfR, ID, and IDA models included adjustment for inflammation (Namaste et al., 2017; Rohner et al., 2017). Health center, maternal education, number of children in the household under 5 years, and minutes between blood draw and aliquot completion were included when *P*<0.1.

[2]Last observations carried forward to cover gaps between observed weeks and from the last recall through the end of 6mo study period

[3]Analysis included children who completed 50% or more of 7-day food frequency questionnaires: ferritin (n=568), sTfR (n=568), Hgb (n=578); anemia (n=578); ID (n=568); IDA (n=561)

Supplementary Figure 1: Participant flow diagram for the iron and dietary analyses of the Mazira Project, Malawi, 2018-2019

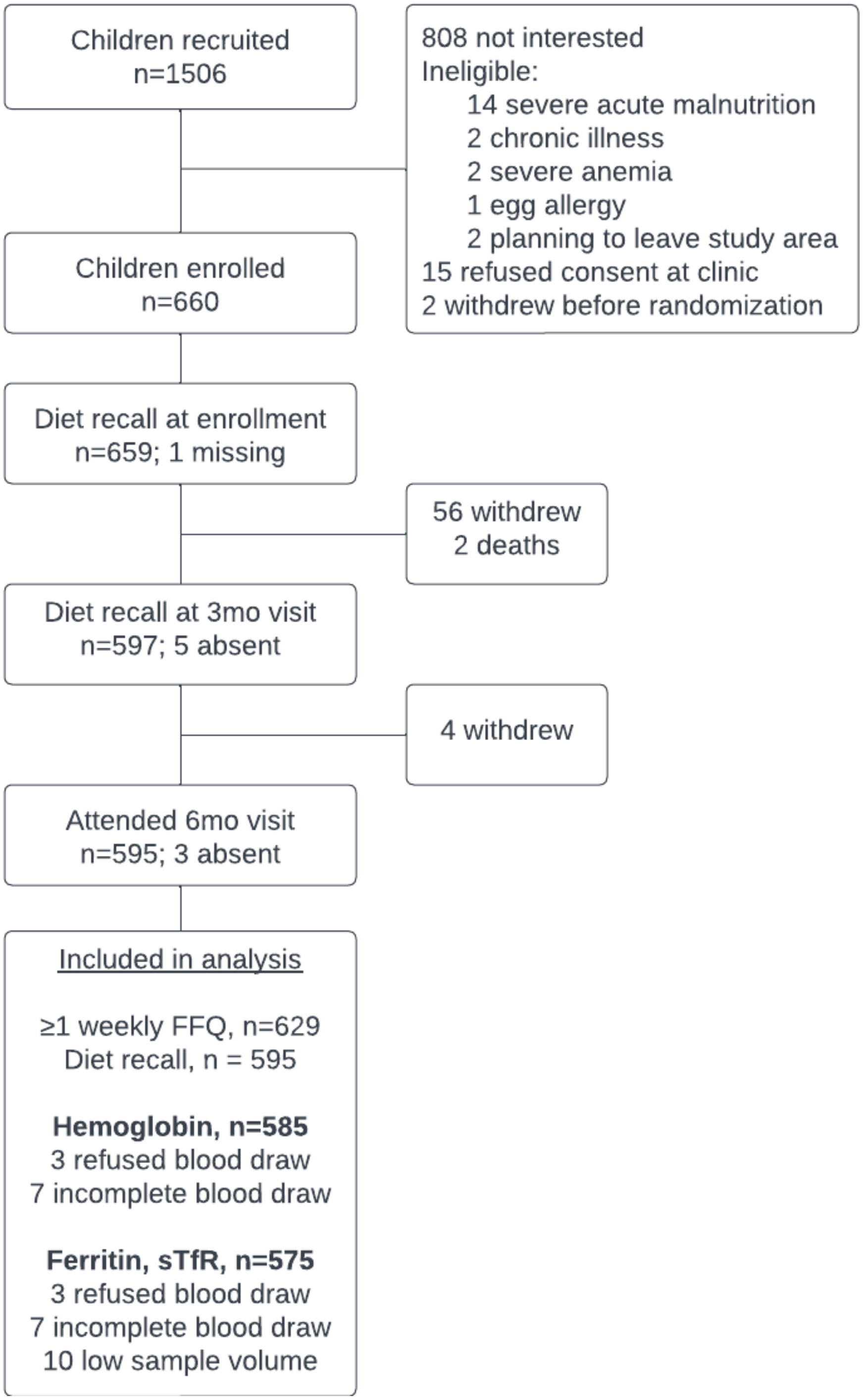

Supplementary Figure 2: Frequency distribution of the percent of days that 6-15mo old children reported consuming meat and fish in Mangochi, Malawi, 2018-2019

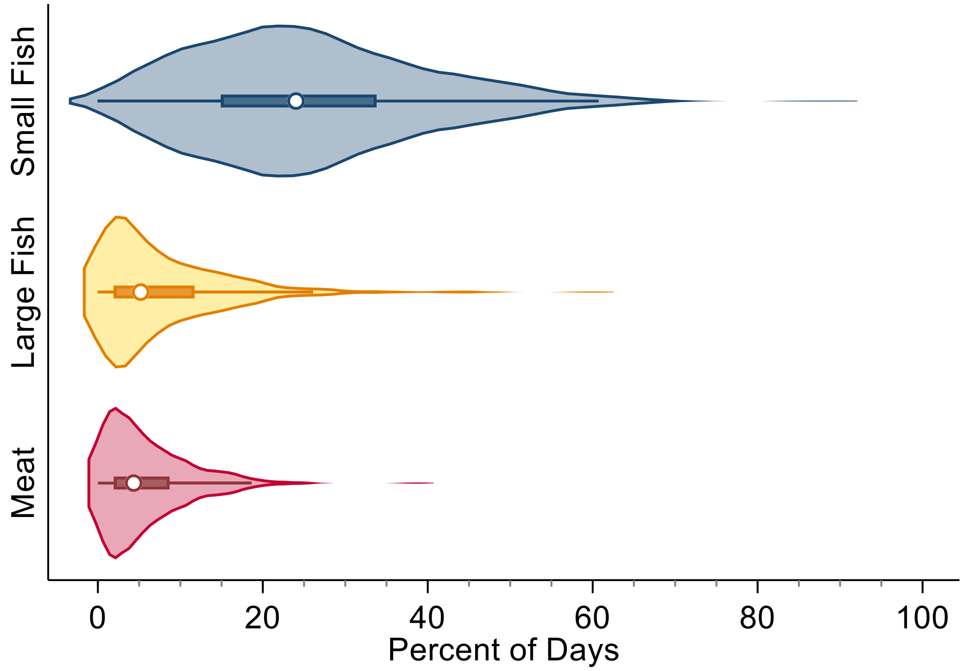

References for Supplementary Material

Callender, S. T., Marney, S. R., & Warner, G. T. (1970). Eggs and Iron Absorption. *British Journal of Haematology*, *19*(6), 657–666. https://doi.org/10.1111/j.1365-2141.1970.tb07010.x

Coates, J., Swindale, A., & Bilinsky, P. (2007). *Household Food Insecurity Access Scale (HFIAS) for Measurement of Household Food Access: Indicator Guide* (3rd ed.). Academy for Educational Development, Food and Nutrition Technical Assistance Project (FANTA).

Cook, J. D., & Monsen, E. R. (1976). Food iron absorption in human subjects: Comparison of the effect of animal proteins on nonheme iron absorption. *The American Journal of Clinical Nutrition*, *29*(8), 859–867. https://doi.org/10.1093/ajcn/29.8.859

Gillooly, M., Bothwell, T. H., Charlton, R. W., Torrance, J. D., Bezwoda, W. R., MacPhail, A. P., Derman, D. P., Novelli, L., Morrall, P., & Mayet, F. (1984). Factors affecting the absorption of iron from cereals. *British Journal of Nutrition*, *51*(1), 37–46. https://doi.org/10.1079/BJN19840007

Hurrell, R.F. (1999). *The Mineral Fortification of Foods*. Leatherhead: Leatherhead International Ltd; pp 54-–93. (As cited in Lynch, S et al. *J Nutr*. 2018; 148:1001S–1067S)

Hurrell, R., & Egli, I. (2010). Iron bioavailability and dietary reference values. *The American Journal of Clinical Nutrition*, *91*(5), 1461S-1467S. https://doi.org/10.3945/ajcn.2010.28674F

Hurrell, R. F., Reddy, M. B., Juillerat, M.-A., & Cook, J. D. (2003). Degradation of phytic acid in cereal porridges improves iron absorption by human subjects. *The American Journal of Clinical Nutrition*, *77*(5), 1213–1219. https://doi.org/10.1093/ajcn/77.5.1213

Petry, N., Egli, I., Zeder, C., Walczyk, T., & Hurrell, R. (2010). Polyphenols and Phytic Acid Contribute to the Low Iron Bioavailability from Common Beans in Young Women. *The Journal of Nutrition*, *140*(11), 1977–1982. https://doi.org/10.3945/jn.110.125369

Namaste, S. M., Rohner, F., Huang, J., Bhushan, N. L., Flores-Ayala, R., Kupka, R., Mei, Z., Rawat, R., Williams, A. M., Raiten, D. J., Northrop-Clewes, C. A., & Suchdev, P. S. (2017). Adjusting ferritin concentrations for inflammation: Biomarkers Reflecting Inflammation and Nutritional Determinants of Anemia (BRINDA) project. *The American Journal of Clinical Nutrition*, *106*(Suppl), 395S-371S. https://doi.org/10.3945/ajcn.116.142307

Rohner, F., Namaste, S. M., Larson, L. M., Addo, O. Y., Mei, Z., Suchdev, P. S., Williams, A. M., Ashour, F. A. S., Rawat, R., Raiten, D. J., & Northrop-Clewes, C. A. (2017). Adjusting soluble transferrin receptor concentrations for inflammation: Biomarkers Reflecting Inflammation and Nutritional Determinants of Anemia (BRINDA) project. *The American Journal of Clinical Nutrition*, *106*(Suppl), 372S-382S. https://doi.org/10.3945/ajcn.116.142232

The Institute of Medicine. (2001). Iron. In *Dietary Reference Intakes for Vitamin A, Vitamin K, Arsenic, Boron, Chromium, Copper, Iodine, Iron, Manganese, Molybdenum, Nickel, Silicon, Vanadium, and Zinc* (pp. 290–393). The National Academies Press.
